# Targeting CXCR4 impaired T regulatory function through PTEN in renal cancer patients

**DOI:** 10.1038/s41416-024-02702-x

**Published:** 2024-05-04

**Authors:** Sara Santagata, Giuseppina Rea, Anna Maria Bello, Anna Capiluongo, Maria Napolitano, Sonia Desicato, Alessandra Fragale, Crescenzo D’Alterio, Anna Maria Trotta, Caterina Ieranò, Luigi Portella, Francesco Persico, Marilena Di Napoli, Salvatore Di Maro, Florinda Feroce, Rosa Azzaro, Lucia Gabriele, Nicola Longo, Sandro Pignata, Sisto Perdonà, Stefania Scala

**Affiliations:** 1 Microenvironment Molecular Targets, Istituto Nazionale Tumori-IRCCS-Fondazione G. Pascale, 80131 Naples, Italy; 2 Urology, Istituto Nazionale Tumori-IRCCS-Fondazione G. Pascale, 80131 Naples, Italy; 3 Department of Hematology, Oncology and Molecular Medicine, Istituto Superiore di Sanità, 00161 Rome, Italy; 4 Department of Neurosciences, Reproductive Sciences and Odontostomatology, Urology Unit, University of Naples “Federico II”, 80138 Napoli, Italy; 5 Uro-gynecological Oncology, Istituto Nazionale Tumori-IRCCS-Fondazione G. Pascale, 80131 Naples, Italy; 6 Department of Environmental, Biological and Pharmaceutical Science and Technology, University of Campania “Luigi Vanvitelli”, 81100 Caserta, Italy; 7 Pathology, Istituto Nazionale Tumori-IRCCS-Fondazione G. Pascale, 80131 Naples, Italy; 8 Transfusion Medicine Unit, Istituto Nazionale Tumori-IRCCS-Fondazione G. Pascale, 80131 Naples, Italy

**Keywords:** Cancer, Renal cancer

## Abstract

**Background:**

Tregs trafficking is controlled by CXCR4. In Renal Cell Carcinoma (RCC), the effect of the new CXCR4 antagonist, R54, was explored in peripheral blood (PB)-Tregs isolated from primary RCC patients.

**Methods:**

PB-Tregs were isolated from 77 RCC patients and 38 healthy donors (HDs). CFSE-T effector-Tregs suppression assay, IL-35, IFN-γ, IL-10, TGF-β1 secretion, and ^Nrp-1+^Tregs frequency were evaluated. Tregs were characterised for CTLA-4, PD-1, CD40L, PTEN, CD25, TGF-β1, FOXP3, DNMT1 transcriptional profile. PTEN-pAKT signalling was evaluated in the presence of R54 and/or triciribine (TCB), an AKT inhibitor. Methylation of TSDR (Treg-Specific-Demethylated-Region) was conducted.

**Results:**

R54 impaired PB-RCC-Tregs function, reduced ^Nrp-1+^Tregs frequency, the release of IL-35, IL-10, and TGF-β1, while increased IFN-γ Teff-secretion. The CXCR4 ligand, CXCL12, recruited ^CD25+PTEN+^Tregs in RCC while R54 significantly reduced it. IL-2/PMA activates Tregs reducing ^pAKT+^Tregs while R54 increases it. The AKT inhibitor, TCB, prevented the increase in ^pAKT+^Tregs R54-mediated. Moreover, R54 significantly reduced FOXP3-TSDR demethylation with DNMT1 and FOXP3 downregulation.

**Conclusion:**

R54 impairs Tregs function in primary RCC patients targeting PTEN/PI3K/AKT pathway, reducing TSDR demethylation and FOXP3 and DNMT1 expression. Thus, CXCR4 targeting is a strategy to inhibit Tregs activity in the RCC tumour microenvironment.

## Introduction

Renal cell carcinoma (RCC) is the third most common newly diagnosed urogenital cancer after prostate and bladder cancer [1]. In 2020, over 400,000 kidney cancer were diagnosed with 180,000 deaths worldwide [2]. Therapy for localised RCC is surgical [3] though 30% presents with metastatic disease and an additional 30% eventually develops recurrence or metastasis [4, 5]. RCC tumour microenvironment (TME) is infiltrated by CD8^+^ T, CD4^+^ T, Natural Killer (NK) cells, macrophages, neutrophils, T regulatory cells (Tregs), and myeloid-derived suppressor cells (MDSCs) [6–8]. Human Tregs, identified as CD4^+^CD25^high^CD127l^ow/-^Foxp3^+^, represent 5–10% of peripheral CD4^+^ T cells. Tregs inhibitory activity depends on cell-to-cell interaction via cytotoxic T-lymphocyte antigen 4 (CTLA-4) and CD28 co-stimulation, secretion of cytokines (IL-10, IL-35), and consumption of T-cell growth factor, IL-2 [9]. Foxp3 locus has conserved noncoding sequences (CNS) [10] targets of epigenetic modifications [11]. Within the FOXP3 enhancer CNS2, Treg-specific demethylated region (TSDR) controls Tregs stability [12, 13]. TSDR is demethylated in natural (n)Tregs, partially methylated in induced (i)Tregs, and completely methylated in effector T cells [14]. In RCC patients, the role of Tregs remains a matter of controversy [7, 15–17] and recently CIBERSORT algorithm correlated Tregs with poorer outcomes [18]. An inhibited PI3K/AKT pathway, negatively regulated by PTEN, is essential for functional and stable Treg cells. PTEN inhibits PI3K and limits phosphorylation of AKT, promoting functional Tregs [19]. Moreover, PTEN is downstream of other Treg-activating signals such as neuropilin-1 (Nrp-1) [20]. Tregs recruitments in the TME depends also on the CXCL12/CXCR4 axis [21]. Cervical cancer, malignant pleural mesothelioma, ovarian cancer and renal cell carcinoma secrete CXCL12, which recruits CXCR4 expressing Tregs [22] impaired by the specific CXCR4 antagonist AMD3100 [23–26]. In ovarian cancer, AMD3100 impaired Tregs function promoting the shift toward CD40L^+^IL-2^+^ T helper-like cells through PTEN loss [26]. A new family of peptide CXCR4 antagonists was developed through a ligand-based approach. A three-residue domain was identified in CXCL12 spatially overlapping with v-MIPII, viral inhibitory chemokine secreted by herpes virus 8 (HHV8) [27] (WO2011092575). Through lead compound optimisation, new powerful analogues, R29 and R54 were developed [28]. R54 displayed subnanomolar affinity toward CXCR4 (IC_50_ ≈ 1.5 nM) and does not bind CXCR3 nor CXCR7 [29]. R29 was previously shown to impair RCC- and HCC- patients derived Tregs suppressive capability [30, 31]. Herein, the mechanism of R54 mediated-CXCR4 antagonism was explored on Tregs from 77 primary RCC patients.

## Patients and Methods

### Patients blood samples

Heparinized peripheral blood (PB) (8 mL) was collected from 77 primary renal cell carcinoma (RCC) patients before partial/radical nephrectomy at the Urology Unit of Istituto Nazionale Tumori di Napoli IRCCS “G. Pascale” and Urology Unit, University of Naples “Federico II”. Heparinized blood was also collected from 38 healthy donors (HDs). The research protocol was approved by the Human Ethical Committee of the Institute (n. CEI/423/13). Clinical features of patients are shown in Table 1.

**Table 1:** Patient characteristics.

	RCC (*n* = 77)
	*N* (%)
**Age (median years)**	
<63	37 (48)
≥63	40 (52)
**Gender**	
Male	46 (60)
Female	31(40)
**Stage**	
pT1	9 (13)
pT2	8 (12)
pT3	15 (22)
pT4	2 (3)
Missing	34 (50)
**Tumour size (cm)**	
≤5	26 (34)
>5	45 (58)
missing	6(8)
**Histological variant**	
Clear cell	51(66)
Papillary	8(10)
Chromophobe	8(10)
Papillary-clearcell	3(4)
Sarcomatoid	1(2)
Missing	6(8)
**ISUP/Vancouver 2012**	
1	5(6)
2	28 (37)
3	12 (16)
4	5(6)
Missing	27(35)

### Migration assay

Tregs migration was assessed as previously described [32–34]. Briefly, freshly isolated peripheral blood mononuclear cells (PBMCs) were obtained from RCC patients and HDs by Ficoll–Hypaque gradients (GE Healthcare Bioscience). 10^6^ PBMCs were stained with APC-Cy7-anti-CD4 and PE-anti-CD25 mAbs to evaluate percentage of CD4^+^CD25^+^ T cell in PBMCs at time 0. Cells were transferred into the upper chambers 5.0 µm pore-size polycarbonate membrane filter 24 Transwell plates (Costar Corning, Cambridge, MA) in a final volume of 200 µL of RPMI-1640 medium (GE Healthcare Life Sciences, HyClone Laboratories). CXCL12 (100 ng/mL; R&D Systems, Minneapolis, MN), was added to the lower chamber and after 16 h the migrated cells were stained with APC-Cy7-anti-CD4 and PE-anti-CD25 mAbs. Migrated cells were counted as CD4^+^CD25^+^ cells lower well/ CD4^+^CD25^+^ cells in upper well X100.

### Purification of T cell subsets

PB samples were processed by Ficoll–Hypaque gradients. Peripheral CD4^+^CD25^+^ Tregs along with peripheral CD4^+^CD25^−^ T effector (Teff) were isolated through Dynabeads Regulatory CD4^+^CD25^+^ T cell kit (Invitrogen by Life Technologies). Briefly, CD4^+^ cells were isolated by negative selection. A depletion beads solution was added to remove the non-CD4 cells. Then, CD25-beads were added to CD4^+^ T cells to capture the CD4^+^CD25^+^ Tregs and the remaining fraction was used as CD4^+^CD25^−^ Teff cells. Finally, Dynabeads CD25 were removed from the cells. Collected cells were >95% pure (confirmed by flow cytometry).

### Tregs suppression assay

Carboxyfluorescein diacetate succinimidyl ester (CFSE)-labelled autologous CD4^+^CD25^−^ T effector cells from PB (CellTrace CFSE Cell Proliferation Kit, Molecular Probes, by Life Technologies) were cultured with peripheral CD4^+^CD25^+^ Tregs at 1:1 ratio. Cells were cultured (5 × 10^3^ cells/well) in U-bottom 96-well plates with RPMI-1640 complete medium (GE Healthcare Life Sciences, HyClone Laboratories). T effector cells were stimulated for 5 days in the presence of Dynabeads Human T-Activator CD3/CD28 (Gibco by Life Technologies). At the end of the coculture, the supernatant and the cells were collected.

### Cytokine assay

ELISA for IL-35, IFN-γ, IL-10, and TGF-β1 was conducted on the supernatant from 5 days suppression tests according to manufacture instructions (Human IL-35 ELISA kit-Boster Biological Technology Human IFN-γ, Human IL-10 and Human LAP (TGF-β1)(Invitrogen-Thermo Fisher Scientific.) Samples were acquired by LB 940 Multimode Reader Mithras (Berthold Technologies).

### Methylation studies: genomic DNA isolation, bisulfite conversion, and qPCR

Methylation analysis was conducted on frozen PB-RCC- and PB-HD-derived Tregs from CFSE-suppression assay. Genomic DNA (gDNA) was extracted and bisulfite treatment of 500 ng genomic DNA was performed by using the EZ DNA Methylation™ Kit (ZYMO Research). qPCR was prepared by using SensiMix SYBR Kit (Bioline, London) and performed by LightCycler^®^ 480 System (Roche Diagnostics). Primers for methylation and demethylation-specific FOXP3-TSDR and computing of the demethylation rate (DMR) of FOXP3-TSDR were previously described [35]. Briefly, we used the following formula: 100/[1 + 2(CtTG- CtCG)] × 100%, where CtTG represents the cycle threshold (Ct) achieved with TG (demethylated) primers and CtCG represents the Ct achieved with CG (methylated) primers. For female patients, this rate was corrected by a factor of 2 since one TSDR allele is methylated as a result of X inactivation [36].

### Real-time PCR

RNA was extracted from frozen PB-HD- and PB-RCC-derived Tregs using the RNeasy Plus Micro kit (Qiagen). Total RNA (1 μg) was reverse transcribed using QuantiTect Reverse Transcription Kit (Qiagen). cDNAs were amplified using CFX96 Touch Real-Time PCR Detection System (Bio-Rad) with iTaq Universal SYBR Green Supermix (Bio-Rad). The primers were designed using the Primer3 tool (http://primer3.ut.ee/). Primers sequence: *CXCR4 Forward:* TGAGGAGCATGACGG, *CXCR4 Reverse:* AGGGAAGCGTGATGA; *DNMT1 Forward:* CGGTTCTTCCTCCTGGAGAATGTCA, *DNMT1 Reverse:* CACTGATAGCCCATGCGGACCA; *TGFb1 Forward:* TGCCCAGAGTGGTTATCTTT, *TGFb1 Reverse:* TAGTGAACCCGTTGATGTCC*; FOXP3 Forward:* AGCACATTCCCAGAGTTCCT, *FOXP3 Reverse:* TGGCGTAGGTGAAAGGGG; *PTEN Forward:* CCAGTGGCACTGTTGTTTCA*, PTEN Reverse:* CCTTTAGCTGGCAGACCACA; *CD25 Forward:* CTGATGTGGGGACTGCTCA, *CD25 Reverse:* GAATGTGGCGTGTGGGATC. Relative expressions of target genes were determined by the 2–ΔΔCt method using 18S and/or B2m as endogenous control. All of the data are presented as means ± sem of three replicate experiments.

### Flow cytometry

After 15 min pre-incubation with Human BD Fc Block Ab (clone Fc1; BD Biosciences) to block Fcγ receptor binding the following antibodies were utilised: APC-Cy7-anti-CD4 (clone RPA-T4), APC-anti-CD25 (clone 2A3), PE-Cy7-anti-CD127 (clone HIL-7R-M2), AlexaFluorV450-anti-Foxp3 (clone 236A/E7), PE-anti-Neuropilin-1 (clone 12C1), BV650-anti-CXCR4 (clone 12G5), anti-APC-CXCR7 (lone 11G8), PE-Cy5-anti-CTLA-4 (clone BNI3), APC-anti-PD-1 (clone MIH4); BB700-anti-CD40L (clone TRAP1), BV605-anti-CD25 (clone 2A3), PE-anti-PTEN (clone A2B1) and AlexaFluor647-anti-pAKT S473 (clone D9E) (BD Biosciences and eBioscience). ^PTEN and pAKT^ Tregs were identified by phospho-intracellular staining according to the BD Phosflow protocol (BD protocol III, https://www.bdbiosciences.com/en-us/resources/protocols/human-whole-blood-samples). Flow cytometry was performed on BD LSR Fortessa X-20 flow cytometer. Data were analysed using FlowJo 10.7 Software.

### Statistical analysis

Fresh peripheral blood samples were prospectively collected before surgery from a total of 77 RCCs and 38 HDs. Available samples during collection were grouped and used for different assays based on a priori sample size calculation for paired and unpaired two-tailed Student’s *t*-test, performed to achieve at least a power ≥80%; given α err prob = 0.05. (G*Power software package; ver. 3.1.9.2; http://www.gpower.hhu.de/Heinrich-Heine-Universität Düsseldorf, Düsseldorf, Germany). Data are presented as mean values±sem. The collected data followed a normal distribution. Two-tailed Student’s *t-*test was the appropriate statistical method used to estimate differences between two independent or matched paired groups of continuous variables. *P*-values less than 0.05 were considered statistically significant (*p < 0.05; **p < 0.01; ***p < 0.001). Variance within groups was not systematically assessed. Data were analysed using the GraphPad Prism 8.0.1 software (GraphPad Software, Inc., San Diego, CA).

## Results

### CXCR4 antagonist R54 impaired PB-Tregs function in RCC patients

**Fig. 1:**
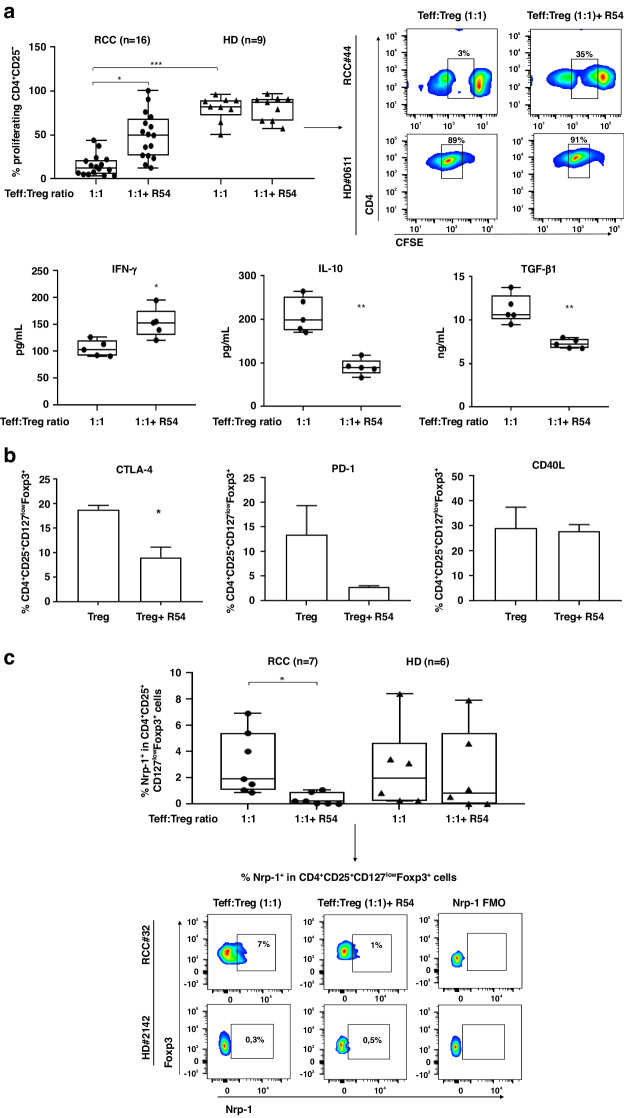
R54 impaired Tregs function in primary RCC patients. **a Upper panel:** CFSE-T effector proliferation in the presence of PB-RCC-Tregs (CD4^+^CD25^+^) and PB-HD-Tregs. Tregs were pretreated for 30’ at 37 °C in 5% CO2 with R54 (10 µM) (RCC: 15 ± 3% in 1:1 vs. 50 ± 6% in 1:1 + R54 Teff: Tregs ratio, *p* < 0.05). The box plot represents the median and spread of data within min to max value (RCC, *n* = 16; HD, *n* = 19). Right panel, representative density plots. **a Lower panel**: IFN-γ, IL-10, and TGF-β1 by ELISA assay in culture supernatant collected on day 5 from CFSE experiments of RCC patients (IFN-γ 1:1 105 ± 7 pg/mL vs. 1:1 + R54 153 ± 12 pg/mL, *p* < 0.05); IL-10 (1:1 210 ± 18 pg/mL vs. 1:1 + R54 90 ± 8 pg/mL, *p* < 0.01); TGF-β1 (1:1 11 ± 0.7 ng/mL vs. 1:1 + R54 7 ± 0.2 ng/mL, *p* < 0.01). The box plot represents the median and spread of data within the min to max value (RCC, *n* = 5). **b** PB-isolated Tregs from RCC patients were evaluated by FACS analysis for frequency of CTLA-4, PD-1, and CD40L (^CTLA-4+^Treg: Tregs 19 ± 1% vs. Treg+R54 9 ± 2%, *p* < 0.05). Histograms represent the mean ± sem (RCC, *n* = 3). **c** ^Nrp1+^Tregs from CFSE assay of RCC patients and HDs (RCC: 3 ± 0.8% in 1:1 vs. 0.3 ± 0.1% in 1:1 + R54 Teff: Tregs ratio, *p* < 0.05). The box plot represents the median and spread of data within min to max value (RCC, *n* = 7; HD, *n* = 6). In the right panel, representative density plots were shown. Paired and unpaired Student’s *t*-test was used. (*p < 0.05; **p < 0.01; ***p < 0.001). Data are derived from at least three independent experiments.

**Fig. 2:**
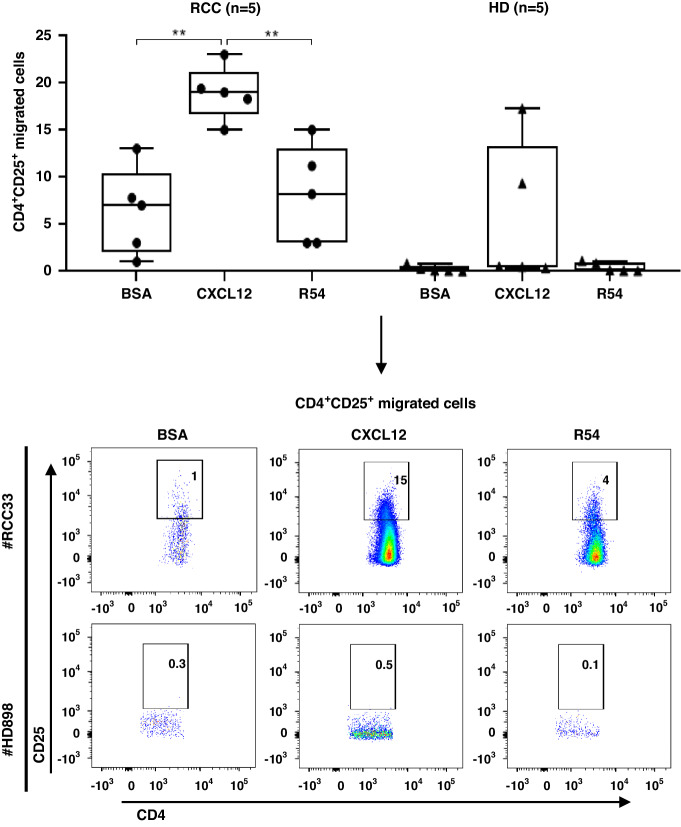
R54 impaired Tregs migration in primary RCC patients. CD4^+^CD25^+^ migration toward CXCL12 (100 ng/ml) plus R54 (10 µM) in RCC patients and HDs (RCC: CXCL12 19 ± 1% vs. BSA: 6 ± 2%; *p* < 0.01; RCC: R54 8 ± 2% vs. CXCL12: 19 ± 1%; *p* < 0.01). The box plot represents the median and spread of data within min to max value (RCC, *n* = 5; HD, *n* = 5). In the lower panel representative experiments were shown. Migrated cells were calculated as CD4^+^CD25^+^ cells lower well/CD4^+^CD25^+^ cells in the upper well X100. Paired and unpaired Student’s *t*-test was used. (*p < 0.05; **p < 0.01; ***p < 0.001). Data are derived from at least three independent experiments.

CXCR4 and CXCR7 expression on Treg and Teff cells was previously reported [30, 37]. Nevertheless, to evaluate the role of R54 on RCC-Tregs, CXCR4 and CXCR7 were evaluated on Tregs and Teff in 5 RCC patients and 5 HDs (Supplementary Fig. S1a–c). As the newly developed CXCR4 antagonist R29 impaired Tregs function [28, 30], we evaluated the efficacy of the most powerful R54 [29]. In Fig. 1a, R54 impaired Tregs suppression of Teff proliferation in PB-RCC-Tregs (*p* < 0.05) but not in PB-HD-Tregs (Fig. 1a, upper panel). According to that, R54 increased IFN-γ (*p* < 0.05) and decreased IL-10 and TGF-β1 (*p* < 0.01) secretion (Fig. 1a, lower panel). Moreover, IL-35 increased in the culture media of PB-RCC-Tregs (*p* < 0.01) and R54 significantly reduced it (Supplementary Fig. S2). Anti-CD3/CD28-Teff stimulation did not affect CXCR4 frequency (Supplementary Fig. S3a) and R54 did not affect Teff proliferation (Supplementary Fig. S3b) in both RCC- and HD-samples. Interestingly, RCC-Tregs treated with R54 displayed lower CTLA-4 (*p* < 0.05) and PD-1 while no significant changes were observed for TH1-helper-like marker CD40L (Fig. 1b). R54 reduced ^Nrp-1+^Tregs [38, 39] in RCC patients (*p* < 0.05) but not in HDs (Fig. 1c, gating strategy in Supplementary Fig. S4). CXCL12-CXCR4 axis was evaluated through CXCL12 dependent-Tregs migration. PB-RCC-Tregs (CD4^+^CD25^+^) migrated toward CXCL12, while PB-HD-Tregs did not, probably due to lower CXCR4 on HD-Tregs. R54 treatment significantly impaired Tregs migration only in PB-RCC (Fig. 2). Thus, R54 impaired PB-RCC Tregs function and reduced peripheral Tregs migration.

### R54 decreased ^CD25+PTEN+^Tregs while inducing ^pAKT+^Tregs in PB-RCC patients

**Fig. 3:**
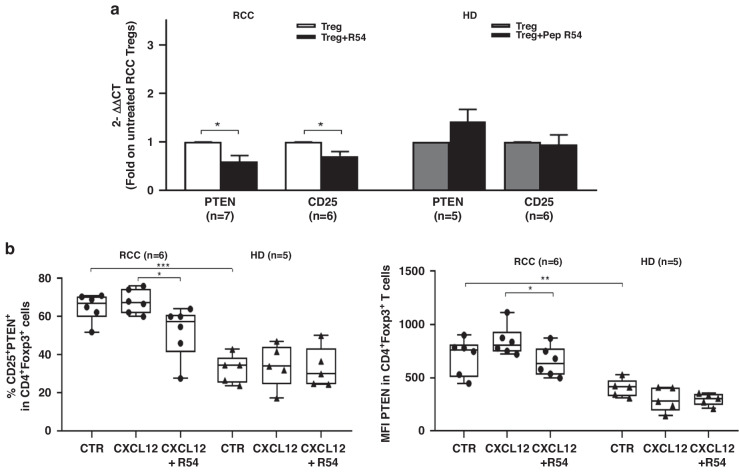
R54 reduced expression of PTEN and CD25 in PB-RCC Tregs. **a** PTEN and CD25 RNA expression in PB-Tregs treated for 30’ with R54 (10 µM) (Treg vs. Tregs+R54, *p* < 0.05). Histograms represent the mean ± sem (PTEN-RCC, *n* = 7; CD25-RCC *n* = 6 and PTEN-HD, *n* = 5; CD25-HD *n* = 6). **b** Flow cytometry ^CD25+PTEN+^Tregs from RCC patients and HD. Isolated-Tregs were treated for 30’ with R54 (10 µM), washed and stimulated with CXCL12 (100 ng/mL) for 2’. In RCC (left): % ^CD25+PTEN+^Tregs CXCL12 treated= 68 ± 3 vs. % ^CD25+PTEN+^Tregs CXCL12 treated+R54 = 52 ± 6%, *p* < 0.05; (right): MFI ^PTEN+^Tregs CXCL12 treated= 846 ± 60 vs. MFI ^PTEN+^Tregs CXCL12 treated +R54 652 ± 59, *p* < 0.05. The box plot represents the median and spread of data within min to max value (RCC, *n* = 6; HD, *n* = 5). Paired and unpaired Student’s t- test was used. (*p < 0.05; **p < 0.01; ***p < 0.001). Data are derived from at least three independent experiments.

**Fig. 4:**
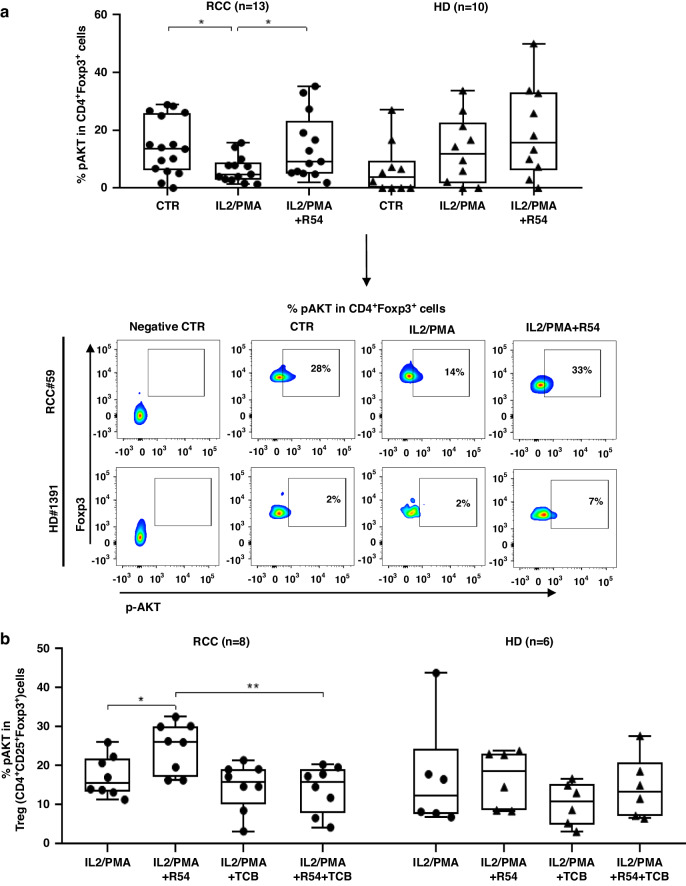
R54 increased pAKT in PB-RCC Tregs. **a** AKT phosphorylation (pAKT) in CD4^+^Foxp3^+^ Tregs in PB-RCC patients and HDs. PBMCs were treated with R54 (10 µM) for 30’, washed and stimulated with IL-2 (100 nM)/PMA (40 nM) for 15’ (RCC: CTR 15 ± 3% vs. IL2/PMA 6 ± 1%; *p* < 0.05; IL2/PMA 6 ± 1% vs. IL2/PMA + R54 14 ± 3%; *p* < 0.05). The box plot represents the median and spread of data within min to max value (RCC, *n* = 13; HD, *n* = 10). In the lower panel, representative density plots were shown. **b** pAKT in isolated PB-RCC-Tregs and HDs in the presence of AKT inhibitors. Tregs were treated for 16 h with AKT inhibitor-(TCB) (20 µM) and then with R54 (10 µM) for 30’, washed and stimulated with IL-2 (100 nM)/PMA (40 nM) for 15’ (RCC: Treg-IL2/PMA + R54 25 ± 2% vs. Treg-IL2/PMA + R54 + TCB 14 ± 2%, *p* < 0.01). The box plot represents the median and spread of data within min to max value (RCC, *n* = 8; HD, *n* = 6). Paired and unpaired Student’s *t*-test was used. (*p < 0.05; **p < 0.01; ***p < 0.001). Data are derived from at least three independent experiments.

To investigate the mechanism through which R54 affects Tregs function, PTEN was investigated [24]. PTEN and α chain of the high-affinity IL-2 receptor (CD25) were evaluated on R54-treated PB-RCC-Tregs. PTEN and CD25 mRNA were significantly reduced in PB-RCC-Tregs (p < 0.05) but not in PB-HD-Tregs (Fig. 3a). Moreover, as shown in Fig. 3b, R54 reduced the percentage of CXCL12 induced ^CD25+PTEN+^Tregs (left panel, *p* < 0.05) and the ^PTEN+^Tregs MFI (right panel, *p* < 0.05) (Fig. 3b and Supplementary Fig. S5a). Although not statistically significant, lower frequency of CD4^+^CD25^+^Foxp3^+^PTEN^+^ was reported in R54-PB-RCC-Tregs in culture with autologous Teff cells (Supplementary Fig. S5b). The effect of R54 was evaluated on AKT, crucial for Tregs activity [19]. Treated R54-PB-RCC and -HDs were stimulated with IL-2/PMA and ^pAKT+^Tregs cells were evaluated. In IL-2/PMA-Tregs a significant reduction of pAKT was detected (*p* < 0.05) while R54 increased it (*p* < 0.05) (Fig. 4a). To confirm that, PB-RCC-Tregs and PB-HD-Tregs were incubated with the AKT inhibitor triciribine (TCB) (20 µM) for 16 h before treatment with R54. Increased frequency of ^pAKT+^Tregs was detected in PB-RCC treated with R54 (*p* < 0.05) while TCB plus R54 reduced the R54 induced ^pAKT+^Tregs (*p* < 0.01). No significant changes in ^pAKT+^Tregs in PB-HD was observed (Fig. 4b). Together, these data suggested that R54 impairs Tregs activity reducing PTEN and increasing pAKT.

### R54 decreased DMR of FOXP3-TSDR and downregulated DNMT1 and FOXP3 in PB-RCC-Tregs

**Fig. 5:**
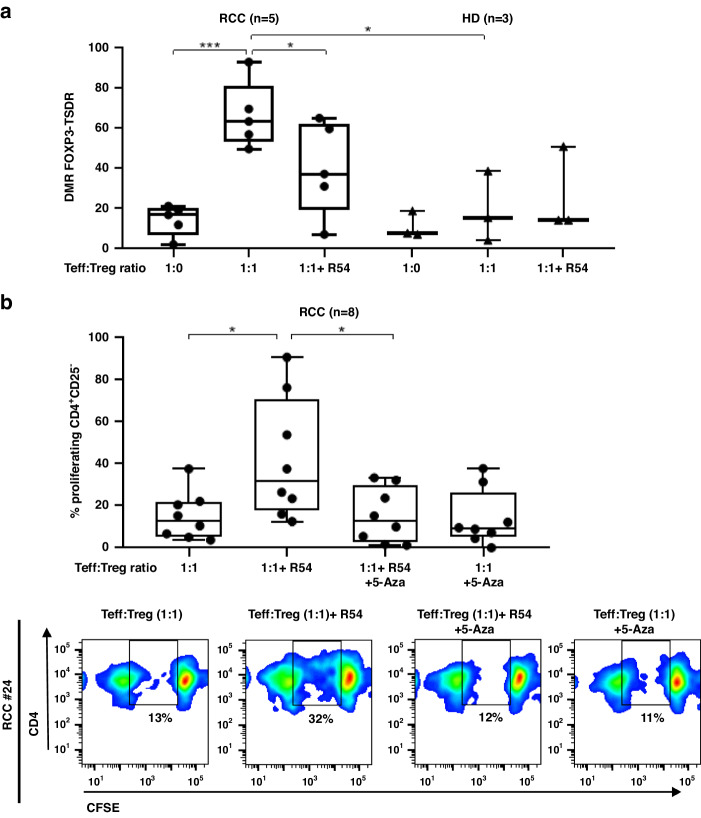
R54 decreased the DMR of FOXP3-TSDR in PB-RCC Tregs. **a** The DMR of FOXP3-TSDR was analysed on R54-PB-RCCs and HDs-bisulfite-converted DNA. Tregs were pretreated with R54 (10 µM) and cultured with autologous Teff from RCC patients and HDs (RCC: 66 ± 6% in 1:1 vs. 40 ± 8% in 1:1 + R54 Teff: Tregs ratio, *p* < 0.05). Box plot represents the median and spread of data within min to max value (RCC, *n* = 5; HD, *n* = 3); **b** 5-Aza reverts R54 inhibition of PB-RCC-Tregs (CD4^+^CD25^+^) were evaluated for suppression of autologous Teff (CD4^+^CD25^-^) proliferation by CFSE assay after 5 days of 1:1 Teff: Treg coculture. Tregs were pretreated for 30’ at 37°C in 5% CO2 with R54 (10 µM), 5-Aza (10 µM) or both (15 ± 4% in 1:1 vs. 42 ± 10% in 1:1 + R54 Teff: Treg ratio, *p* < 0.05; 42 ± 10% in 1:1 + R54 vs. 15 ± 5% in 1:1 + R54 + 5-Aza, *p* < 0.05). The box plot represent the median and spread of data within the min to max value (RCC, *n* = 8). In the lower panel, representative density plots were shown. Paired and unpaired Student’s *t*-test was used. (*p < 0.05; **p < 0.01; ***p < 0.001). Data are derived from at least three independent experiments.

**Fig. 6:**
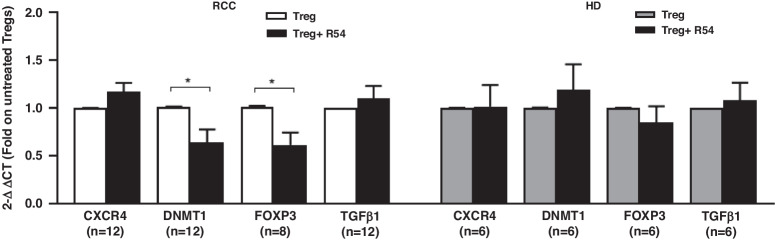
R54 decreased the expression of DNMT1 and FOXP3 in PB-RCC Tregs. Real-time PCR for isolated PB-RCC-Tregs (CD4^+^CD25^+^) and PB-HD-Tregs. RNA was extracted from frozen RCC and HD derived-Tregs pretreated with R54 (10 µM) for 30’(RCC: DNMT1 and FOXP3 Tregs vs. Tregs+R54, *p* < 0.05). Relative expressions of target genes were determined by the 2–ΔΔCt method using 18S and/or Beta2 microglobulin as endogenous control. Histograms represent the mean ± sem (CXCR4/DNMT1/TGF-β1-RCC, *n* = 12; FOXP3-RCC, *n* = 8; CXCR4/DNMT1/TGF-β1/FOXP3-HD, *n* = 6). A paired Student’s *t*-test was used. (*p < 0.05; **p < 0.01; ***p < 0.001). Data are derived from at least three independent experiments.

**Fig. 7:**
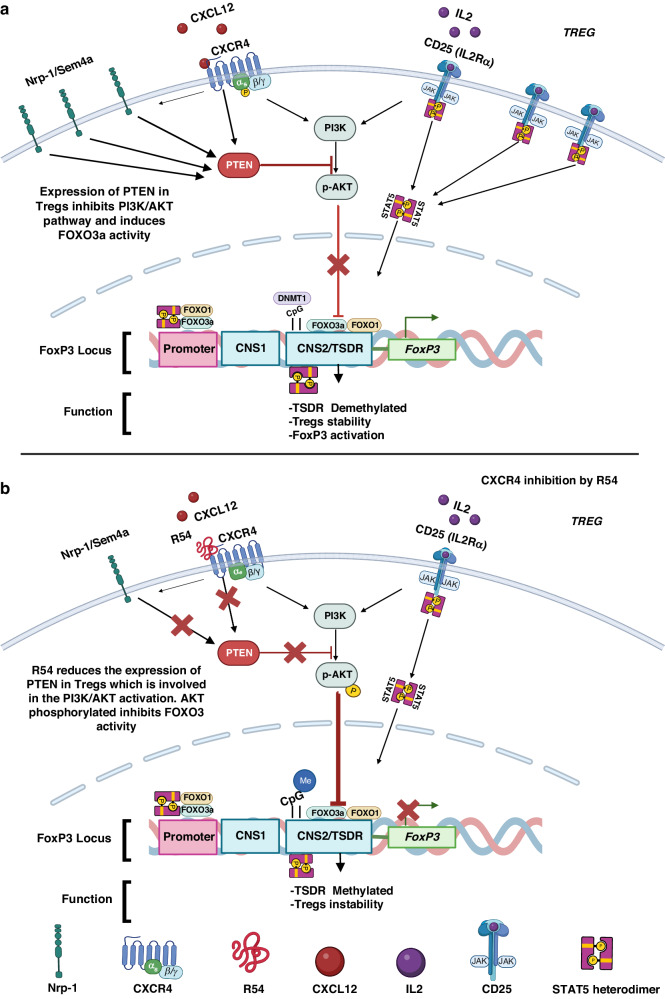
Schematic of R54 effect on Tregs signalling pathways. **a** CXCR4/CXCL12 and CD25/IL2 activates PI3K, pAKT. Surface receptors such as Nrp-1 and CXCR4 activate the PTEN phosphatase, which inhibits PI3K activity with consequent low pAKT. DNMT1 (CpG islands) and FOXO3a/FOXO1 transcription factors controlled the demethylated/methylated status of the Treg-specific demethylated region (TSDR), within the Foxp3 locus. Without AKT activity, FOXO1 and FOXO3a are active and induce Tregs stability, TSDR Demethylation, and then the transcription of the FOXP3 gene. **b** R54-mediated CXCR4 inhibition in Treg cells results in reduced surface Nrp-1 and CD25 and reduced PTEN. The absence of PTEN, a negative regulator of the PI3K/AKT signalling pathway, induces the PI3K/AKT activation resulting in pAKT that inhibits FOXO3 and FOXO1 activity. Activated AKT phosphorylates and inactivates the transcription factors FOXO3a and FOXO1, contributing to TSDR Methylation and Tregs instability which causes downregulation of the Treg suppressor phenotype (Created with BioRender.com).

Functional Tregs present demethylation of TSDR within the FOXP3 locus [12, 14, 40]. Previously, high DMR of FOXP3-TSDR was reported in suppressive PB-Tregs from RCC patients [30]. Herein, we analysed the effect of R54 on FOXP3-TSDR methylation in Tregs from PB-RCC and PB-HD. After 5 days of coculture with autologous Teff, a significant decrease in DMR of FOXP3-TSDR was observed in R54-treated PB-RCC-Tregs (*p* < 0.05). Conversely, R54 did not affect the DMR of FOXP3-TSDR in Tregs from HD (Fig. 5a). Tregs function increased with the demethylating agent 5-Azacitidine (5-Aza) treatment [41]. Herein, R54 efficiently reversed RCC-Tregs mediated suppression of Teff proliferation (p < 0.05) while the demethylating agent 5-Aza reverted the R54 effect (*p* < 0.05); thus, R54-Tregs, potentiated Teff proliferation and the addition of 5-Aza to R54 reduced it, confirming R54-mediated functional Tregs impairments (Fig. 5b). As consequence of TSDR modulation, the expression of Tregs regulating genes (TGF-β1, FOXP3, DNMT1 and CXCR4) were tested in R54 treated PB-RCC patients and PB-HDs. R54 significantly decreased the expression of DNMT1 and FOXP3 in PB-RCC-Tregs (*p* < 0.05) but not in PB-HD-Tregs (Fig. 6). No significant changes were observed in Teff from both R54-PB-RCC patients and PB-HDs (Supplementary Fig. S6). In the graphical abstract, the described results are recapitulated (Fig. 7).

## Discussion

In this manuscript, the efficacy of the new CXCR4 antagonist, R54, was evaluated on peripheral Tregs isolated from 77 primary renal cancer patients. R54 impaired PB-RCC Tregs function decreasing PTEN and potentiating AKT signalling. PB-RCC Tregs were more frequent and suppressive in RCC patients as compared to healthy donors. Higher IL-35, immunosuppressive, and anti-inflammatory cytokine [42] was reported to increase Tregs tumour recruitment promoting immunosuppression [43, 44]. IL-35, TGF-β, and IL-10 Tregs suppress the activity of APCs and Teff cells [45] while ^CTLA-4, PD-1^Tregs promote the suppressive function [46]. Herein, R54 impaired PB-RCC-Tregs function reducing frequency of ^Nrp-1+^Tregs/^CTLA-4+^Tregs, reducing immunosuppressive cytokines (IL-35, IL-10, and TGF-β), T effector proliferation, Tregs migration, DMR of TSDR and FOXP3 and DNMT1 transcription. PTEN signalling plays a crucial role in this process. During PI3K-AKT activation, PI3K phosphorylates the membrane phospholipid PIP2, generating PIP3, which acts as an anchor for AKT. Full active AKT is phosphorylated at S473 and T308 by the kinases mTORC2 and PDK1. PTEN opposes PI3K-AKT activation by catalysing the reverse reaction, dephosphorylating PIP3 into PIP2 [47]. Active Tregs exhibit comparable PI3K activity but dampened AKT activation, particularly at the mTORC2-dependent S473 phosphorylation site [48, 49]. Low AKT activity is functionally relevant in Tregs, as overexpression of constitutively active AKT inhibits mouse Tregs development in vivo and in vitro [19, 49–51]. Small molecule AKT activator, SC79, impaired the growth of poor immunotherapy responder murine tumours, B16 and EMT-6 suppressing CD4^+^Foxp3^+^Treg TILs through the conversion of Tregs to IFNγ^+^CD4^+^Th1-like T cells [52]. Nevertheless, PTEN can play a double, opposite role: as a powerful tumour suppressor in tumours cell and as an immune suppressor in Tregs [53–55]. PTEN absence results in reduced CD25 expression, the accumulation of Foxp3^+^CD25^-^ cells, and ultimately, the loss of Foxp3 expression [56]. Downstream of PTEN, reduced AKT activation maintains FOXO transcription factor activity, which is needed for Tregs development and function [57]. CD4, CD8, and DCs-expressed ligand semaphorin-4a and the Tregs expressed Nrp-1 potentiates in vitro Tregs stability and function. Particularly, semaphorin-4a ligation of Nrp-1 restrained AKT phosphorylation via PTEN, which increased nuclear localisation of the transcription factor FOXO3a [20]. FOXO1 and FOXO3 promote Foxp3 expression, upregulate CTLA-4 and other Treg-associated genes, and inhibit the expression of the inflammatory cytokine IFN-γ [57–59]. TSDR methylation in Foxp3^+^CD25^+^ and Foxp3^+^CD25^−^ cells purified from wild-type and PTEN-ΔTreg mice revealed a moderate reduction of TSDR demethylation in CD25^+^ PTEN-deficient Tregs compared to wild-type CD25^+^Tregs [56]. Herein, R54 reduced Foxp3 and DNMT1 transcription, as previously reported in murine ovarian cancer, where the CXCR4 inhibitor, AMD3100, selectively reduced intratumoral Foxp3 Tregs [23] and DNMT1 determining loss of Tregs suppressive function in vitro and in vivo [56, 60]. Several evidence demonstrate a transcriptional link between CXCR4 and PTEN activity focusing on AKT. In osteosarcoma, PTEN loss activates AKT/CXCR4 signalling while in vivo PTEN overexpression correlates with reduced CXCR4 expression [61]. In colon cancer, CXCL12 induced transcriptional down-regulation of activated PTEN promoting cell survival [62]. Consequently, the downstream targets of PI3K/AKT, Nuclear factor κB (NF-κB), and activator protein 1 (AP-1) can be abnormally activated [63, 64]. In ovarian cancer, CXCR4 was detected among 681 hypo methylated-upregulated genes while PTEN, and FOXO-1 were mentioned among the hypermethylated repressed genes [65] and in vascular smooth muscle cell (SMC), vascular injury increased PTEN/AKT signalling through CXCL12- HIF-1alpha [66]. As NFkB is a target of the CXCR4-CXCL12 pathway [67, 68] and HIF-1alpha controls CXCR4 transcription [69] it is possible to speculate that the transcriptional link between the axis CXCL12-CXCR4 relies on HIF-1alpha, NFkB and/or methylation processes.

In conclusion, the CXCR4 antagonist R54 reduces CD25 and PTEN expression on PB-RCC Tregs, resulting in a pAKT increase and, thus, a defect of Tregs activity. Taken together, our findings demonstrated that R54 causes impairment of peripheral Tregs in primary RCC patients through regulation of the PTEN/PI3K/AKT pathway, reduction in TSDR demethylation and Foxp3 and DNMT1 expression. CXCR4 targeting is a strategy to inhibit Tregs activity contributing to the CXCR4 function in the RCC tumour microenvironment.

### Supplementary information

Supplementary Figures

Supplementary Figure Legends

## Data Availability

The datasets used and/or analysed during the current study are available from the corresponding author on reasonable request. Datasets are available at 10.5281/zenodo.10419111.
