# Supplementary material for: Targeting CXCR4 impaired T regulatory function through PTEN in renal cancer patients
Source: Br J Cancer. 2024 May 4;130(12):2016–26. doi: 10.1038/s41416-024-02702-x (PMC11183124; doi:10.1038/s41416-024-02702-x)

Supplementary Figure S1

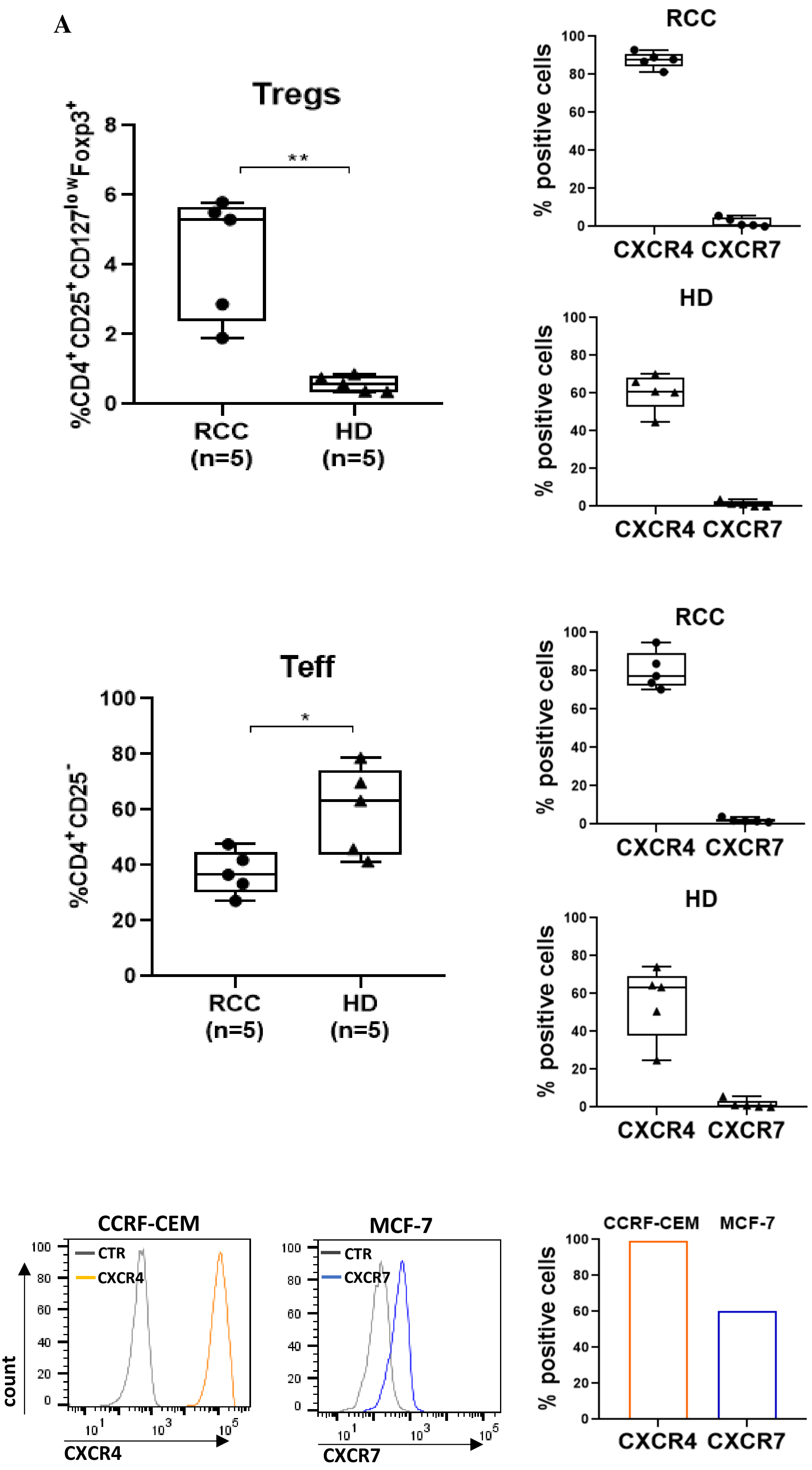

Supplementary Figure S1

B

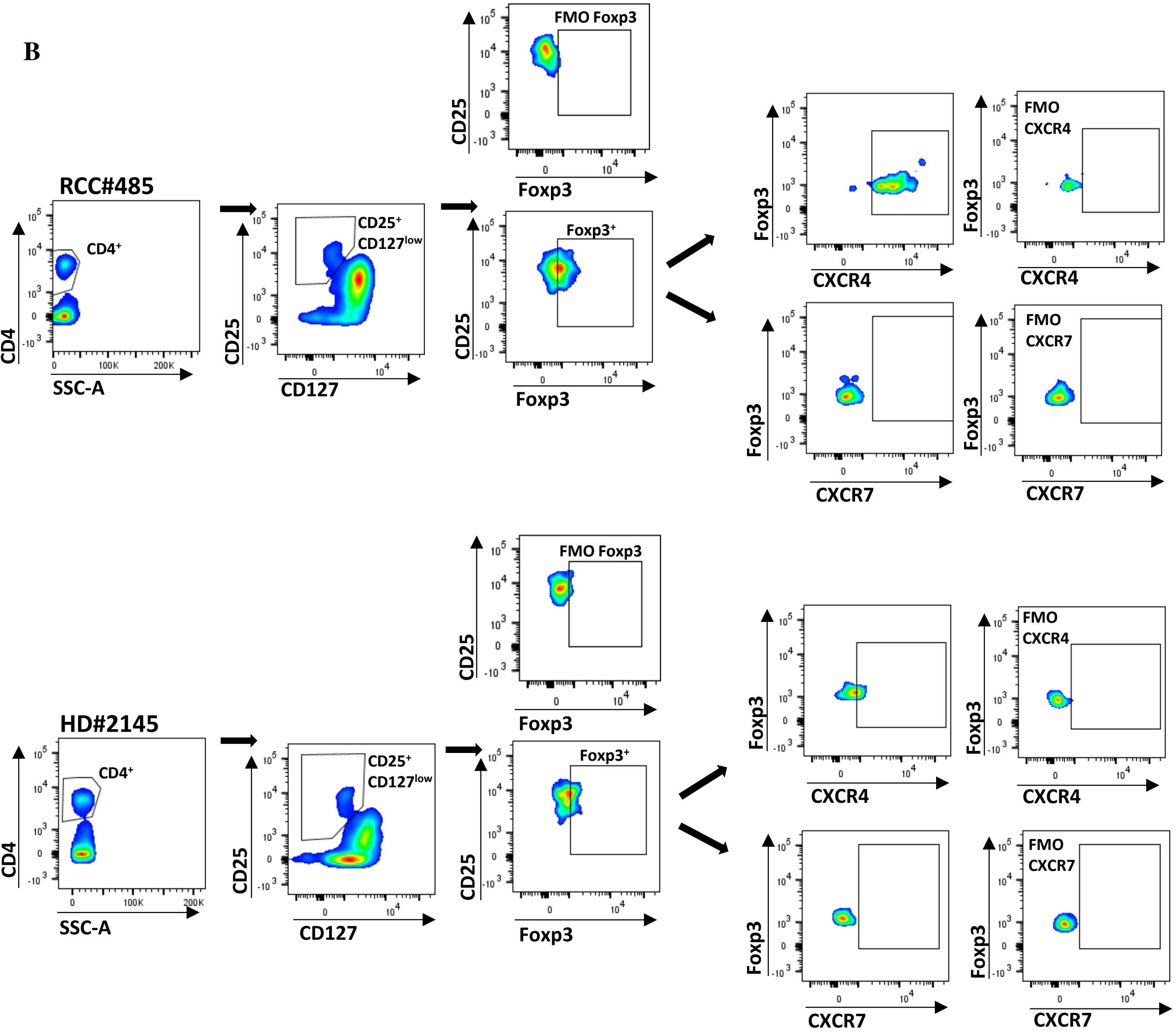

C

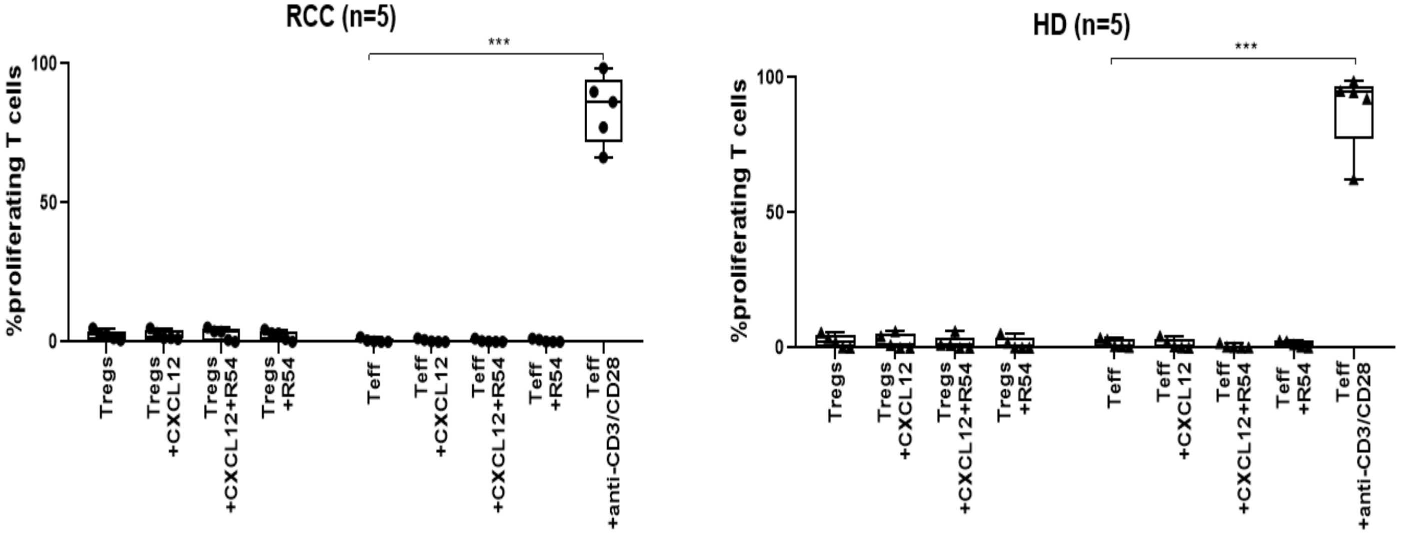

Supplementary Figure S2

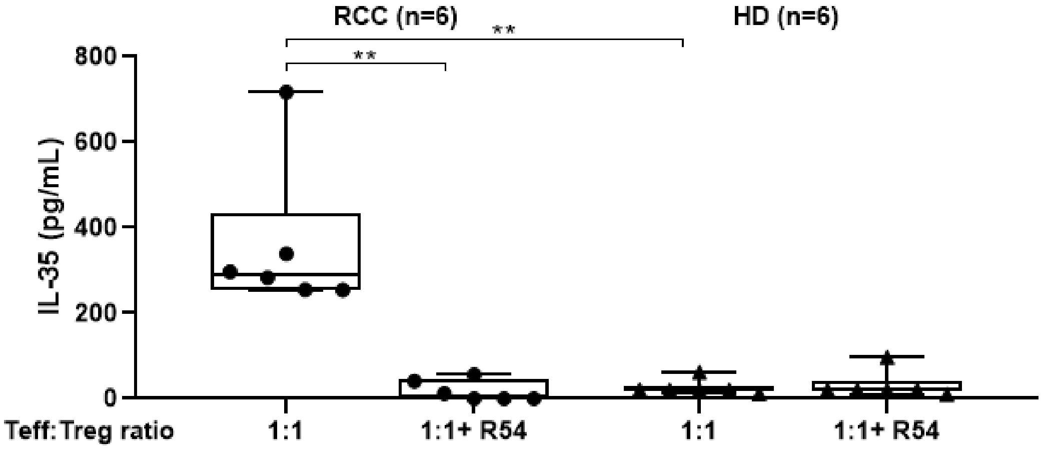

Supplementary Figure S3

A

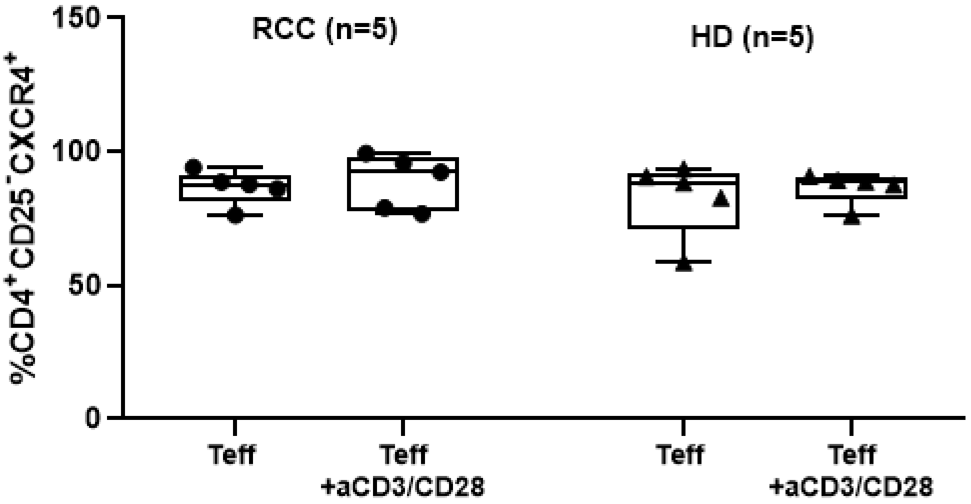

B

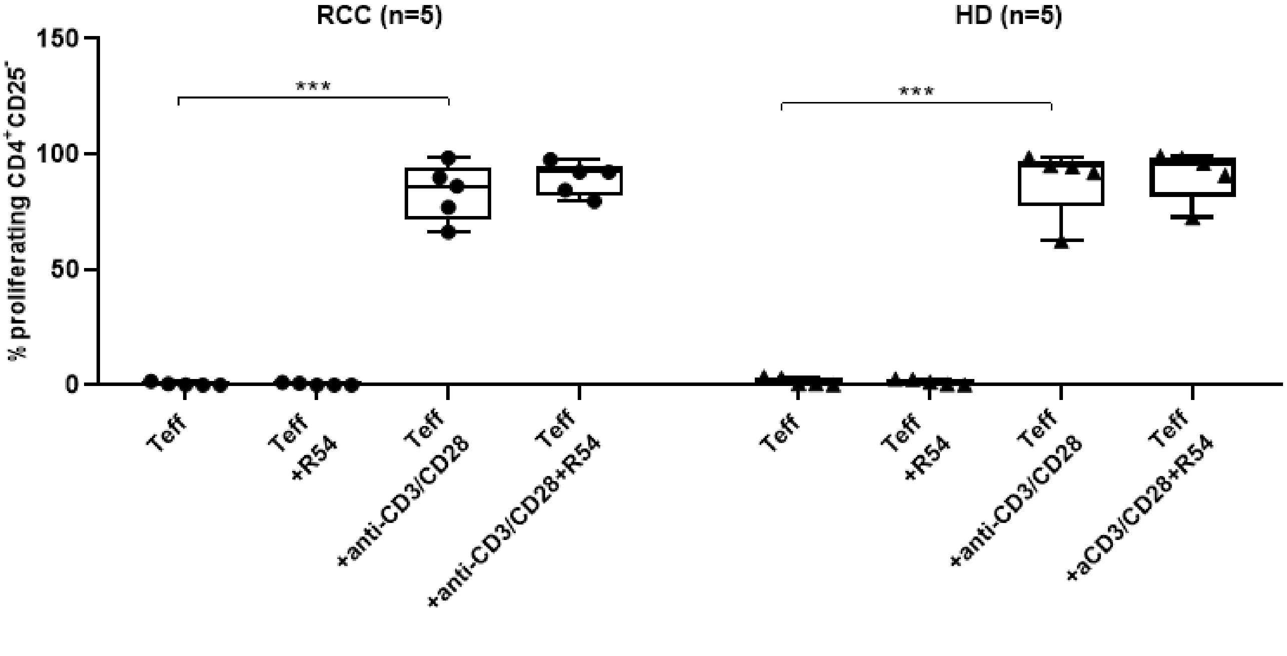

Supplementary Figure S4

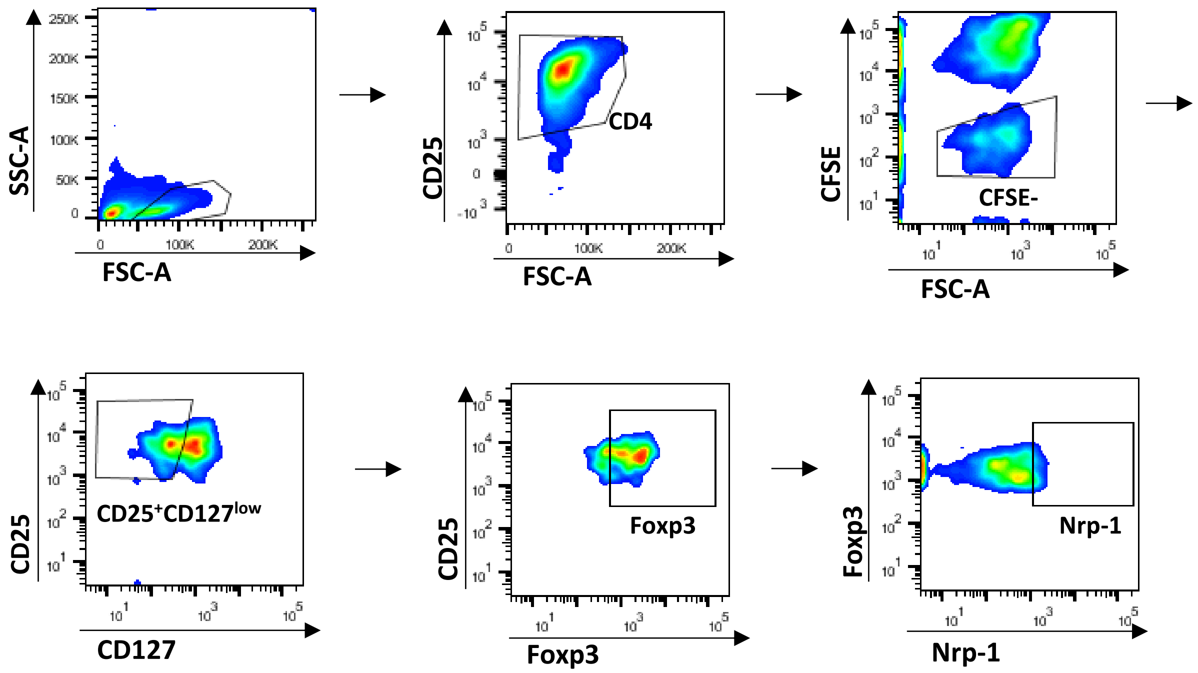

Supplementary Figure S5

A

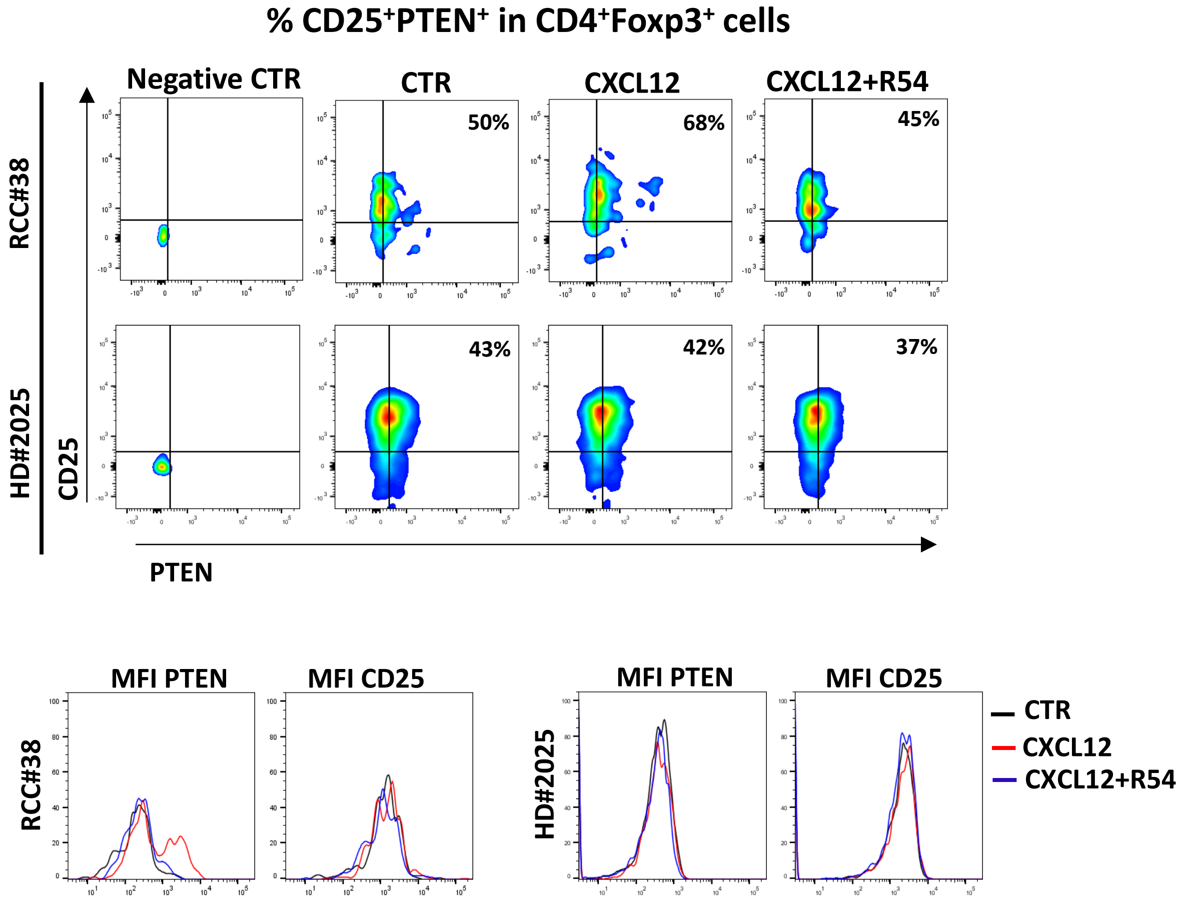

B

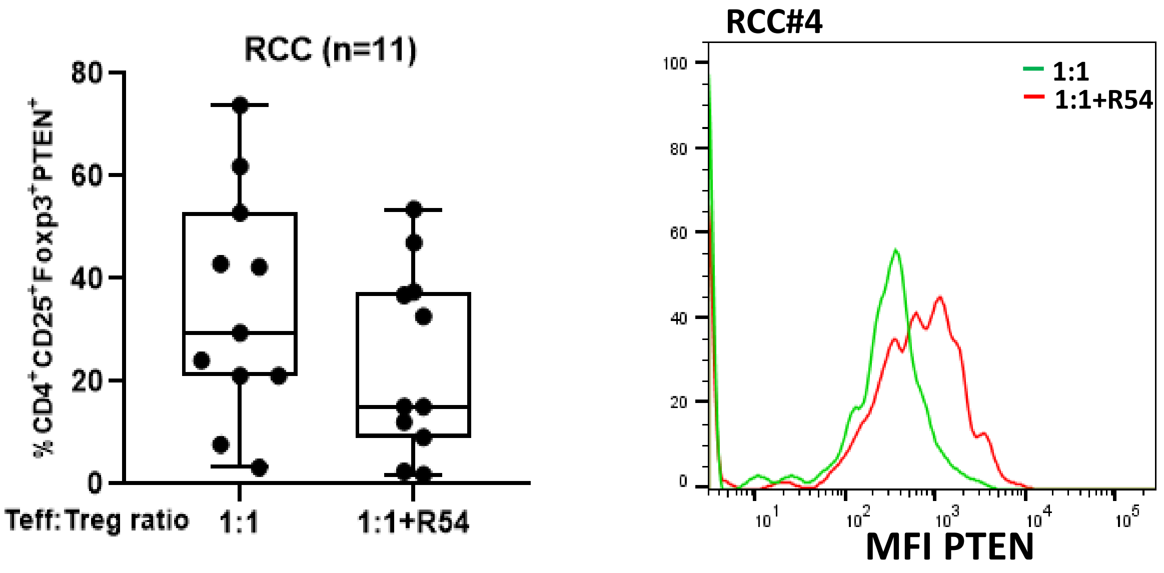

Supplementary Figure S6

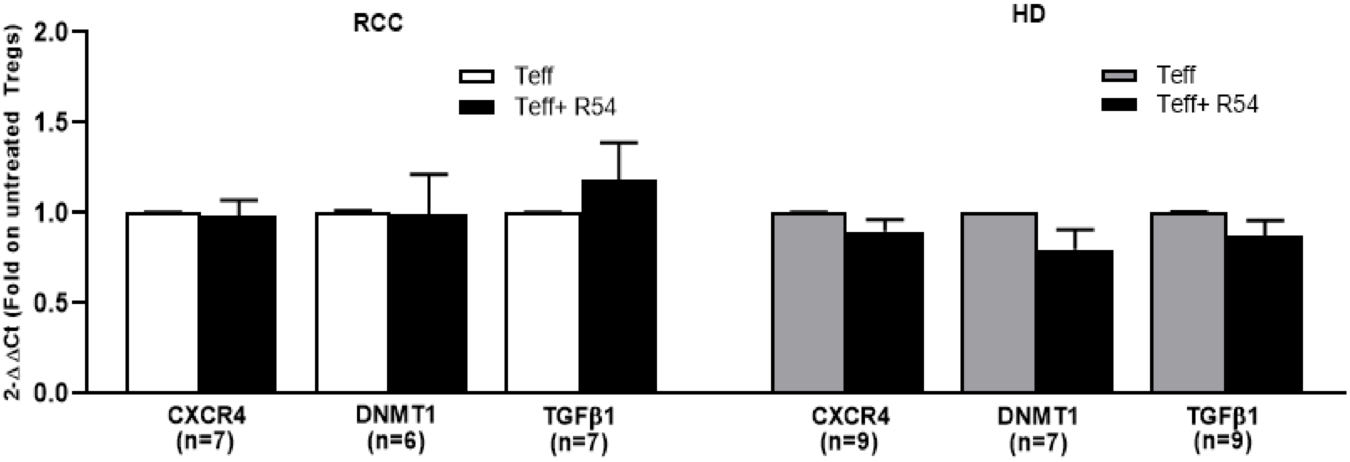

Supplement: Supplementary file 1 — Supplementary Figures [file 41416_2024_2702_MOESM1_ESM.pdf]
