# Supplementary material for: Targeting CXCR4 impaired T regulatory function through PTEN in renal cancer patients
Source: Br J Cancer. 2024 May 4;130(12):2016–26. doi: 10.1038/s41416-024-02702-x (PMC11183124; doi:10.1038/s41416-024-02702-x)
Supplement: Supplementary file 2 — Supplementary Figure Legends [file 41416_2024_2702_MOESM2_ESM.docx]

**Supplementary Figure S1. CXCR4 and CXCR7 frequency in Tregs and Teff from RCC patients and HDs.**

**(A)** Frequency of ^CXCR4+^ or ^CXCR7+^Tregs (CD4^+^CD25^+^CD127^low^Foxp3^+^) and ^CXCR4+^ or ^CXCR7+^Teff (CD4^+^CD25^-^) in the peripheral blood of RCC patients and HDs by flow cytometry (CXCR4 on RCC-Tregs: 87.6±1.9%; CXCR4 on HD-Tregs: 60.9±4.3%; CXCR7 on RCC-Tregs: 2.2±1%; CXCR7 on HD-Tregs: 1.2±0.6%; CXCR4 on RCC-Teff: 80±4.3%; CXCR4 on HD- Teff: 55.4±8.5%; CXCR7 on RCC- Teff: 2±0.5%; CXCR7 on HD- Teff: 1.6±1%). CCRF-CEM and MCF-7 cells considered as positive control cells for CXCR4 (CCRF-CEM 99%) and CXCR7 (MCF-7 60%). **(B)** Representative gating strategy for CXCR4 and CXCR7 analysis was shown. **(C)** CFSE-proliferation of isolated Tregs or Teff induced by CXCL12 in RCC patients and HDs. Isolated cells were treated with R54 (10µM) for 30’ and then kept in culture for 5 days with CXCL12 (100ng/mL). As a positive control, Teff cells were stimulated with anti-CD3/CD28. Box plot represents the median and spread of data within min to max value (RCC, n= 5; HD, n=5). Paired and unpaired Student’s t- test was used. (*p< 0.05; **p< 0.01; ***p< 0.001). Data derive from at least three independent experiments.

**Supplementary Figure S2.** **R54 decreased IL-35 secretion in RCC-Tregs.** IL-35 ELISA in culture supernatant collected on day 5 from CFSE experiments in RCC patients and HDs (RCC: 357±73 pg/mL in 1:1 vs 18±10 pg/mL in 1:1+R54, p<0.01). Box plot represents the median and spread of data within min to max value (RCC, n= 6; HD, n=6). Paired and unpaired Student’s t- test was used. (*p< 0.05; **p< 0.01; ***p< 0.001). Data derive from at least three independent experiments.

**Supplementary Figure S3. RCC patients- and HD-anti-CD3/CD28 stimulated Teff express comparable CXCR4. Teff proliferation is unaffected by R54. (A)** Frequency of CXCR4 on isolated Teff (CD4^+^CD25^-^) stimulated with anti-CD3/CD28 in RCC patients and HDs by flow cytometry. **(B)** CFSE-proliferation of isolated Teff in RCC patients and HDs. Isolated cells were treated with R54 (10µM) for 30’ and then kept in culture for 5 days with anti-CD3/CD28. Box plot represents the median and spread of data within min to max value (RCC, n= 5; HD, n=5). Paired and unpaired Student’s t- test was used. (*p< 0.05; **p< 0.01; ***p< 0.001). Data derive from at least three independent experiments.

**Supplementary Figure S4**. **Gating strategy of Nrp-1 on Treg cells**. Flow cytometry analysis of Nrp-1 on CD4^+^CD25^+^CD127^low^Foxp3^+^ Tregs (in CFSE negative cells) post CFSE-coculture assay.

**Supplementary Figure S5. R54 decreased ^PTEN+^Tregs in RCC patients**. **(A)** Representative density plots of ^CD25+PTEN+^Tregs and PTEN/CD25 MFI histograms were shown from RCC patient and HDs. **(B)** PTEN expressing RCC-Tregs with or w/o R54 in CFSE-proliferation assay by flow cytometry (35±10% in 1:1 vs 14±7% in 1:1+R54). Box plot represents the median and spread of data within min to max value (RCC, n= 11). In the right panel, a representative overlay plot was shown. Paired Student’s t- test was used. (*p< 0.05; **p< 0.01; ***p< 0.001). Data derive from at least three independent experiments.

**Supplementary Figure S6. R54 effect on CXCR4, DNMT1 and TGFβ1 expression in Teff from RCC and HD**. CXCR4, DNMT1 and TGFβ1 RNA expression in isolated Teff treated for 30’ with or w/o R54 (10 µM) by Real-time PCR. Histograms represent the mean±sem (CXCR4/TGFβ1-RCC, n=7, DNMT1-RCC, n=6; and CXCR4/TGFβ1-HD, n=9, DNMT1-HD, n=7). Paired Student’s t- test was used. (*p< 0.05; **p< 0.01; ***p< 0.001). Data derive from at least three independent experiments.
